# Gleanings from Our Exchanges

**Published:** 1872-10-15

**Authors:** 


### Gleanings from Our Exchanges.

### THE BRITISH MEDICAL ASSOCIATION.

The following cases from the address in Midwifery, delivered by Evory Kennedy, M.D.—a continuation of those given in our last—are taken from the excellent abstract of the proceedings of the late meeting of the Association, given in the September and October numbers of *The Doctor:*

*The Whole Circle of the Os Uteri Thrown Off in Labor.*—A patient in her third labor, which was somewhat violent, had slight hæmorrhage. The head was well engaged in the pelvis, and some fleshy mass was found to protrude from the vulva. The pains increasing, a complete circular ring, about three-eighths of an inch thick by an inch in breadth, escaped, which, on examination, proved to be the detached os uteri in its entirety. The labor proceeded rapidly. There was very little hæmorrhage; and the patient made a favorable recovery, the lochiel discharge continuing longer and being more offensive than usual. Of the after-history of this case, and whether she bore more children, I am ignorant.

*Acephalous Fœtus; totally brainless; acutely sensitive.*—A female child, otherwise fully formed and developed, was born acephalous—in fact, totally devoid of brain. It survived its birth one hour and a half, breathing and crying loudly ; the expirations were rather convulsive in their character. Its voice was very strong. It was acutely sensitive to impressions made on the surface of the body, and moved its limbs. We could not satisfy ourselves whether it heard or saw. It cried so much on inserting food in its mouth, that it was impossible to ascertain whether it could perform the act of deglutition. In other cases of acephalous fœtuses, we usually found a rudimentary portion of the middle lobe of the brain; and they rarely survived beyond a few gasps, if so much. The malformation is occasionally combined with spina bifida and contortions of the limbs; but some, like this child, are fully developed—indeed, the shoulders were so much so as to create a difficulty in their extraction. The *post-mortem* examination, in which I was assisted by the late Dr. Todd, of King's College, presented no feature worth recording, beyond what I have mentioned.

*Spina Bifida.*—In June, 1872, I was consulted about a child four months old, reasonably well developed, healthy, and with every prospect of continued vitality and health, save the existence of a head somewhat large, but with tardy development of the parietal and frontal bones, leaving the large fontanelle unossified, congenital dislocation of the ankles forwards, and a spina bifida of the lumbar vertebræ. This measured two inches in diameter, with a projecting polished membranous covering, enclosing an elastic fluctuating sac. In fact, there was a total absence of the integumentary, ligamentous, and bony covering of the spinal cord, the thickened dura mater supplying their place. I exhibit a model of this case. The child, a girl, shows quite as much evidence of mind and powers of observation as four months' children generally do. My directions hitherto have been to take every precaution to prevent the injury or rupture of the cyst. A chambered shield of gutta percha, lined with padded silk, is being adapted to prevent the possibility of accidental injury; and I have it in contemplation to try a plastic operation when the age, promise of enduring life, and condition of the child justify it. It would be extremely gratifying to me to have the suggestions of my *confreres* with reference to the future dealing with this remarkable case; more especially of those who have met with similar cases in their practice. How long have they been known to survive, and how they were treated?

*Vesico-vaginal Fistula cured by twisted Suture.*—On August 12th, 1837, H. Byrne, æt. 25, was sent from the country suffering from vesico-vaginal fistula. The opening was longitudinal, about five lines long, with considerable loss of substance. It was about three inches from the urethral orifice. The bowels were well freed. She was placed on the abdomen on the edge of a table, with a bolster interposed. My four curved beak-tractors were introduced, and, her limbs being held fast by assistants, I pared the edges of the fistula with a scalpel, cutting on an ebony spatula; and, having introduced three short needles into the wounded edges, drew them together with a twisted suture. A perforated shot was now squeezed on the end of each needle, an elastic catheter introduced into the bladder, and the patient was placed lying on her abdomen, on bolsters stiched together longitudinally. She was retained in this position, and closely watched day and night, by a relay of nurses, who never left her. The urine flowed freely from the urethra into a bladder attached to the catheter. her bowels were washed out by lavement. Not a drop of urine escaped from the vagina. The sutures were cut out on the fifth day, and the needles removed, when the union was complete. She left the hospital in a fortnight perfectly well. I had the advantage of the assistance of my late colleague, the distinguished surgeon Mr. Abraham Collis, in this case; and I cannot easily forget the gratification he evinced when he made a final examination of the bladder before the patient left the hospital, nor the significant manner in which he answered my inquiry whether he could find any hole. "No, doctor; I can't find a hole, unless I make one;" and added, with a peculiarly expressive shrug of his shoulders, with which those who knew him were familiar, "I am sure you do n't wish me to do that."

*Vesico-vaginal Fistula treated by Cautery and Pessary.*—Mary Cathcart (November, 1837) suffered from a slough of the vesico-vaginal septum in her first labor in the country. The fistula was small, circular, about two inches and a half from the meatus, and rather to the right side. This was considerably diminished by one application of the actual cautery a month after her third delivery; but as this was followed by peritonitis, it was determined not to repeat any operation. A cast model of the vagina, made of dentist's wax, was taken. This was done by introducing the soft wax, with a tape for extraction imbedded in its center, through a speculum partially introduced. A caoutchouc mould was made from this, which fitted the vagina accurately, and was worn constantly as a pessary. The result of this simple contrivance, to use the words of my then clinical clerk, copied from this report, was, that "she experienced much comfort, being enabled by its use to retain her urine to a convenient length of time, and evacuate the contents of the bladder at pleasure, without withdrawing the instrument." I should, however, mention that I have since several times repeated this plan, using the cautery in preference to the ligature, or where this was objected to; and it has generally enabled the patient to retain her urine for some time. It has, however, not always been attended with the same amount of success. In fact, it is more adapted to very small fistulous apertures, or to those that have been reduced by the use of the cautery.

*Total Occlusion of Vagina: Operation: Animation suspended for upwards of twenty-two Minutes by Chloroform.*—Some years ago, Dr. Ringland and I were called upon to operate in a case of a lady with total occlusion of the vagina. The examination could only be made through the rectum. The anterior and posterior walls of the vagina were united almost throughout their whole length. There was a sense of fluctuation perceptible on pressing the finger high up into the bowel. A tumid fullness was perceptible over the pubes, to about the extent of a six months' pregnancy. The bladder and rectum were emptied; and the patient was placed on her back, on a high couch, with the limbs flexed and held by assistants, as in lithotomy. The patient was with some little difficulty brought under the influence of chloroform. I had commenced the operation, when she struggled so that I was obliged to desist, and assist in bringing her completely under its influence, before again commencing. A catheter was introduced into the bladder, and the dissection was proceeded with, cutting and separating the adherent tissues with the blade and handle of the scalpel alternately; now introducing the finger into the rectum, and again feeling through the anterior wall for the catheter, to avoid approaching too closely to either cavity — a scarcely appreciable divergence in either direction being fraught with misery to our patient for life. From the difficulties and risk attending the operation, it was necessarily a tedious one ; " rapidity " in such an operation being a convertible term for " destruction." At length, after cutting for three inches upwards, and dissecting a new vaginal canal by dividing throughout this extent a layer of condensed structure not more than the sixth of an inch in thickness, we had the gratification of coming to a small pouch at the upper part of the vagina, which communicated with the distended uterus, and from which poured a quantity of grumous retained menstrual fluid. Having thoroughly enlarged the opening at the upper part, so as to correspond with the dimensions of the canal throughout, I got up from the operation to receive a shock such as I never experienced before or since. Our attention was called by the chloroformist to our patient, who lay with her head over the edge of the table, her jaw fallen, and to all appearance dead. There was no respiration; no pulse at the wrist; no action of the heart. I took out my watch, in order to take care that attempts to restore animation should be continued for a sufficient time before desisting, but without a hope that they could be attended with success. Experience in resuscitation of infants had led me to expect little from forcible inflation of the lungs by inserting a tube into the trachea, but much from a continuous and persistent imitation of the act of respiration by regular pressing on the elastic ribs of the subject—producing, as nearly as possible, the systole and diastole of the lungs and chest-frame, as observed in nature. Without a moment's delay, I sprang upon the high table, so as to command the prostrate woman ; and, kneeling across her, placed a spread hand over the lower ribs, and kept up an artificial respiratory action in the lungs of about twenty pressures in a minute. In the meantime, all the available means of resuscitation were most assiduously carried out by my friend Dr. Ringland and his assistants. Friction and sinapisms were applied to the arms, legs and surface. As speedily as buckets of warm water could be procured, her hands, feet, and limbs were immersed in it. These efforts were persisted in, whilst minute after minute was anxiously counted, with not the slighest evidence of restored vitality. At length, our souls absolutely sickened with disappointment, and, I may add, all hope having fled, we were, at the expiration of twenty-two minutes, repaid for our exertions by a convulsive gasp. Nearly a minute took place before a second occurred. Then they recurred at half-minute intervals; and eventually the natural breathing became established, and the artificial respiration was desisted from. Sickness now set in. She was unable to assist in the involuntary efforts to discharge the stomach. The food blocked up the œsophagus and posterior fauces; but, by drawing the head and neck over the edge of the table, and giving her the advantage of gravity whilst the finger was pressed into the œsophagus, the obstructing food and mucus were removed, and respiration was established. A second collapse ensued after a few minutes, consequent on the evacuation of the uterus; but after this she recovered without a check. A curious circumstance, in a psychological point of view, should not be omitted. When she recovered her sensibility and powers of perception and speech, within a few minutes after respiration was re-established, looking steadfastly at one of the physicians present, she asked slowly and with some effort, " Is that Jesus Christ?" Where was her soul during that period of suspended animation ? It might be surmised, that this case would shake one's confidence in chloroform. With me, it had an entirely contrary effect, as it proved the power — perhaps I should say, the capability — of restoring vitality suspended by its use under circumstances apparently hopeless.

*Obstinate Procidentia of Uterus Cured by Actual Cautery.* — Mary Burke, æt. 60 a widow with six children, had obstinate procidentia of the uterus, and could not retain any form of pessary. She suffered much distress; her general health was deranged; she was dyspeptic, and had lumbar pains. She was confined to the horizontal position, with the lips well raised, for a fortnight. The ulcers healed. The uterus was placed *in situ*, and astringent injections were used. The actual cautery was then applied about an inch and a half from the vulva round the surface of the vagina for the extent of about half an inch. My clinical clerk, who was somewhat of the Gil Blas school, in criticising his master, adds that the old woman was so indignant at this operation, which, for obvious reasons it was deemed more considerate not to explain beforehand, that she left the hospital in a fit of indignation, because a hot iron had been applied to her inside, as she said, without saying, " With your leave, or by your leave." She eventually permitted the treatment to be followed up by the application of nitrate of silver; and the report is thus continued by my clinical critic. At the expiration of two months, she was able to go about again. The uterus was *in situ:* and she had derived much benefit to her general health and condition, and a distressing pain, descending down the front of the thigh, from which she had constantly suffered, had disappeared. The cicatrix formed by the eschar prevented the descent of the os. He adds, however, this caustic comment on my operation: "She certainly got a regular touching up."

### MYALGIA AND ITS TREATMENT.

BY J. B. MATTISON, M. D., OF CHESTER, N. J.

Myalgia, strictly speaking, is the term applied to those very common and painful affections, grouped under the head of muscular rheumatism, and known as lumbago, pleurodynia, and so forth, according to the muscle or muscles affected.

Doubtless, every practitioner is more or less familiar with these troubles, and though some of them may be comparatively trivial, as, for instance, the stiff neck produced by cold, and, being treated with domestic remedies, escape his professional attention, there are others, in which the actual suffering, and incapacity for physical labor are so marked, and so protracted, that it becomes a matter of prime importance to remedy this state of affairs as speedily and effectually as possible.

It is not the purpose of this article to speak of the pathology of this disease, nor of its diagnostic points as compared with neuralgia, both features of marked importance, and for a full consideration of which the reader is referred to Dr. Anstie's admirable treatise on "Neuralgia and its Counterfeits," the latest and best work on the subject, and with which every physician should thoroughly acquaint himself, if he be desirous of keeping fully abreast of the times in the management of that painful affection.

We desire more particularly, to call attention to a plan of treatment, which, in our hands, in *all* cases, has given more prompt and satisfactory results than any other with which we are acquainted, namely, the subcutaneous injection of morphine.

Anstie, in speaking of the treatment of this affection, remarks, that keeping the affected muscle or muscles in a state of full extension, the maintenance of a perpetual local vapor-bath by spongio-piline, and, if necessary, one or two Turkish-baths, are all that is required in a very large number of cases. When these measures fail, he recommends the use of scruple or half drahm doses of muriate of ammonia, and adds, that "in the first very acute stage of a severe case it may be advisable to inject morphia hypodermically."

Now, the point we desire to bring prominently forward is, that it is not necessary to wait for a *severe* case, but that in *all* cases where professional assistance is desired, the hypodermic employment of morphia, varying the dose according to the intensity of the suffering, and in the milder cases a very small quantity will suffice, affords more decided, and by far more speedy relief than any other mode whatever.

The result in the following cases will fully illustrate the value of this form of medication.

March 13, 1872, was called upon to visit R. P., farmer, æt. 45, whom I found confined to his bed from a severe attack of lumbago of four day's duration. Any attempt to assume an upright position gave rise to the most exquisite suffering, and even when the affected muscles were completely extended and fully at rest, there was an intense aching, entirely preventing sleep, and causing much distress.

At the cost of great pain we succeeded in getting him in a semi-prone position, and injected one-third of a grain morphine sulphas over the lumbar region. In seven minutes, *actual time*, patient was free from pain, and expressed himself as feeling perfectly comfortable. Visited him the next day, 12 M. Complete relief, with no unpleasant effect save a slight nausea, had continued for sixteen hours, allowing a night's refreshing slumber, and only returned, very much lessened; about an hour previous to my second visit. Injected one-quarter grain sulphate, and that completed the treatment, as the next day the patient left his bed, and the second day afterwards reported himself in the village three miles distant from his residence. He was most emphatic in his testimony as to the value of the remedy, and declared that after the first injection "he was never so happy in all his life."

H. M., æt 17, presented himself at the office, complaining of pain in the left side, much increased on full inspiration, and of several weeks' standing. An examination into the history of the case convinced us that it was myalgic, and an injection of gtt. v. solut. magend. was followed, in five minutes, by entire relief from pain, *which did not return.*

W. H., æt. 35, applied for the full relief of a pain of four months' duration, presenting the following peculiarity: When erect, either sitting or standing, no discomfort was felt, but the moment he bent forward, or elevated his feet above the level of his body, he began to suffer pain in the epigastrium, which constantly increased in severity until it compelled him to resume an upright position. He presented the appearance of perfect health, and a careful examination failed to discover the slightest evidence of gastric derangement. Regarding the case as one of epigastric myalgia, we injected gtt. viij. sol. magend. in the affected muscle. Saw the patient some hours after, and he expressed himself very much relieved, only slight pain being felt in any position. Circumstances of a business nature beyond control prevented his following out the plan of treatment, and he passed from under our observation. We have no doubt that the injections, had they been continued, would have resulted in a perfect cure.

Was called to visit Mrs. T., æt., 29, mother of seven children, the youngest eleven months old. The following is her history. Two weeks subsequent to the birth of her last child, she was seized with a severe pain under her left breast, which has occurred at variable intervals ever since. Not a day has passed without more or less distress, so marked, at times, as to compel her for a while, to abandon domestic duties. Her general health seemed good and were it not for the local suffering she would consider herself a well woman. There was a sense of soreness in the affected region, and the pain seemed to be dependent, in a great measure, upon muscular action, movements involving the affected part always distinctly agravating it. Sinapisms and other remedies had been employed without benefit. Considering the case myalagic in its nature, we injected gtt. iv. sol. magend. deeply into the substance of the painful part. Called forty-eight hours aftarwards, and learned the injection gave marked and permanent relief, having felt no pain since its employment, an immunity from suffering she had not enjoyed for nearly a year. Made a second visit some time later, and found the freedom from suffering continued. Meanwhile. she had engaged in more than ordinarily severe labor without any pain whatever, and having no further occasion for professional services after the case was dismissed.

The foregoing are not culled cases. They are given in the order they occurred, and the results in all, are, we think, such as to verify the statement made as to the value of the remedy employed.

Some doubts may be expressed as to the correctness of diagnosis in the last mentioned case, from the fact that one injection sufficed for a cure. The absence of any other manifestation of hysteria, past or present, will exclude the so-called "hysterical breast," while the want of any neurotic tendency in the previous history, and the fact that the pain was always distinctly agravated by muscular movement, will, we think, set aside any suspicion as to its neuralgiac nature.

While claiming for the subcutaneous employment of morphia the decided preference over other forms of treatment, we by no means deny that cases may occur requiring additional measures. It is pre-eminently in those instances where the affection is strictly local that its curative virtues are so marked. When any evidence of anæmia exists, and the pain seems but part of a general malnutrition, tonic remedies, and especially the continued employment of cod-liver oil, will be quite essential to effect a radical cure.

One point, in particular, we desire should not be overlooked, namely, *deep* injections. Plunging the needle firmly into the substance of the affected muscle, gives us the benefit of *acupuncture*, which has been found exceedingly valuable in getting rid of obstinate myalgic pain.—*Phil. Med. Reporter*.

### ALCOHOL AS A COOLING REMEDY.

In the papers on "therapeutic traditions" in course of publication in the *Lancet*, the writer has been treating of "cooling remedies." He thus speaks of alcohol in this respect:—

With regard to alcohol, the substantial agreement (*a*) of nine-tenths of the observers who have studied the subject may be considered, even in opposition to a single illustrious objector—Dr. Parkes—to prove that the influence of alcohol upon temperature except when this is subnormal, is uniformly in the direction of *lowering*, particularly when it is given in narcotic doses. [The present writer has reason to add, from his own researches on this subject, that moderate doses of alcohol, compare, on the whole, very closely, in their effects on the temperature of healthy persons, with that of ordinary food; that the latter not unfrequently appears to cause a fractional *fall of temperature;* that alcohol (in ordinary dose) does this a little more decidedly, but not constantly; that, on the other hand, alcohol in narcotic dose produces a very substantial lowering influence. Indeed, he is strongly inclined to believe that the following statement, paradoxical as it

(*a*) Cunibert Bouvier (Pharmakologische Studien uber Alkohol; Bonn, 1871) has marshalled this evidence with striking effect.

sounds at first hearing, is literally true; that the more swiftly oxidisable a food is, the less likely is it to give occasion to the direct development of free heat, and the more likely to produce a positive fall of the thermometer when given in excessive quantity in health. In this view alcohol, for healthy persons, would simply be regarded as the most oxidisable of hydro-carbonaceous foods, though unfortunately, it has other properties, of a destructive kind, when taken in continued excess.]

Such being the state of our knowledge with regard to the action of food and alcohol in health, we have now to consider both theory and observed fact as regards their influence on *morbid excess* of temperature. In pyrexia, as is well known, the incidence of the bodily chemical changes is greatly altered: the elimination of nitrogen proceeds as before, but is now derived in great part from the destruction of nitrogenised tissues instead of the destruction of nitrogenised food; and this kind of process results in the development of a far larger proportion of energy *in the condition of free heat* than is produced by the conversion of food in time of health. Under these circumstances there appears no theoretic reason why food, provided it be readily oxidisable, should not form an efficient protection to the tissues, and a consequent depressor of febrile heat; and in truth, such objections to its use as really exist are mainly accidental—*e. g.*, certain foods cannot practically be given because the fevered stomach refuses to do the work of primary digestion upon them. Two forms of food, however, are rarely embarassing to primary digestion in pyrexia, except where the stomach is itself inflamed—viz., milk and alcohol. The almost unlimited use of milk is possible in many forms of pyrexia ; and milk, either alone or combined with starch (in the form of arrowroot, &c.), frequently affords a striking instance of a food which lowers morbid temperature. The present writer has frequently seen (as others doubtles have) a typhoid fever patient experience a remarkable reduction of temperature and simultaneous amelioration of general condition upon the administration of this kind of nourishment. The effects of alcohol in the same direction are even more remarkable (*a*), but they are not constant; some mysterious law determines differences which are at present ultimate facts, incapable of solution. When alcohol acts without narcotising it reduces morbid temperature ; but whether it will narcotise or not, and if so in what dose, are points as to which we can never be certain without experimental trials; although a certain speed and a certain form of pulse-wave, together with the presence of delirium, will frequently direct us rightly. Much yet remains quite unknown as to the profounder relations of alcohol to the fevered organism and the causes which allow it to be tranquilly oxidised in large quantities by one fever patient, while much smaller doses will disorder the system of another. But the fact remains that for pyrexial patients, presenting a certain group of phenomena which are now fairly well known, alcohol produces the truest and the most direct reduction of temperature—with the one exception of that caused by the cold bath—of which we have any example in the whole round of known remedies.

We have thus dwelt on the susceptibility of alcohol to oxidation, because that phase of its action assimilates it to common food. Our readers are well aware, however, that this is not the only probable source of the heat-lowering power of alcohol. The supposed action upon small cellular bodies which Binz, more especially, has assigned to it, is one extremely probable source of this cooling power.—*The Doctor.*

(*a*) Dr. Ringer probably underrates the amount of the reduction that can be thus effected, at any rate in the most appropriate cases.

THREE CASES OF EPILEPSY. — Dr. L. P. Yandell, Jr., reported to the College of Physicians and Surgeons, the following cases, the first of which possesses unusual interest as having resisted the action of the bromide of potassium alone, but was rapidly benefitted and finally relieved by the addition of the sulphate of atropia to the prescription.

Case 1. A girl, aged thirteen years, small, delicate, precocious, has had since early childhood, excrutiating headaches, attended by vomiting, double vision, and sometimes loss of consciousness and delirium, and always sending her to bed for a day or more. The seizures were sometimes excited by undue heat or cold, excesses in eating or exercise, malarial trouble or menstrual derangement. She had been treated by bromide of potassium in full doses for many months without relief. When patient came under Dr. Y's care he gave her from twenty to sixty grains of the bromide three times daily, and one-hundredth of a grain of the sulphate of atrophate of atropia night and morning. The attacks soon became less frequent and less severe, and in seven months she seemed rid of them altogether. She was directed, however, to continue the medicine for twelve months more. The patient has of late had several attacks of genuine hemicrania, which were entirely rebellious to the bromide and atropia, but which promptly disappeared under quinine.

Case 2. A young farmer, whose father, a confirmed epileptic, died hemiplegic and insane, had many convulsions during infancy and early childhood. Just after reaching puberty he had, while working in the hot sun, his first epileptic seizure. Has had attacks for four years pretty regularly every month, sometimes oftener. A course of bromide of potassium and sulphate of atropia, with strict attention to all his habits of life, have now averted a paroxysm for upwards of a year. He takes the medicine still, though in diminished doses.

Case 3. A Swede, aged twenty-seven, an epileptic since early youth, having attacks almost every month, was usually warned of the approach of the seizures by a distinct aura beginning in one hand. He was not unfrequently able to avert the attack by clinching the fist of this side with all his force, or laying violent hold on some hard substance, as a table or chair. The same treatment pursued in the previous cases materially improved the patient, and gave decided promise of curing him, when he left the hospital, and has not since been heard from. Dr. Y. has come to regard epilepsy as curable in the large majority of cases by the bromide of potassium and sulphate of atropia. —*Med. Reporter.*

Mixed Vapors for Anæsthesia.—Dr. J. D. Davis (*Medical Record*), in commenting upon this subject, remarks that the mixture recommended by the Committee on Anæsthetics, of the American Medical Association, deserves to be more universally known and used, its advantages being—the small quantity required; the rapidity of its action; the shortness of the stage of excitement (it often being wanting entirely); the absence of distressing after effects; its comparative safety over chloroform; being compounded in definite proportions, it can be scientifically administered; the pungent, stifling odor of the ether and the burning sensation of the chloroform, are to a great extent overcome; the sedative effects of chloroform are modified by the exciting influence of ether, while to both is added the stimulant alcohol. The mixture is composed of alcohol, one part; chloroform, two parts; and ether, three parts. It is easily remembered by taking the initials in the order of the alphabet: A., C., E., the corresponding number of parts being 1, 2, 3.

External Use of Fuming Nitric Acid.—Professor Patruban (*Memorabilien*, June, 1872), says that this heroic caustic is indicated on account of its rapidity and drying-up of the tissues. It stops all effusion of blood, and leaves good scars. He uses it for warts and other protruberances of the skin, as condylomata, epulis, hæmorrhoids, and tumors of the nature of nævus.—*The Doctor.*

